# Mutational landscape of inflammatory breast cancer

**DOI:** 10.1186/s12967-024-05198-4

**Published:** 2024-04-18

**Authors:** François Bertucci, Florence Lerebours, Michele Ceccarelli, Arnaud Guille, Najeeb Syed, Pascal Finetti, José Adélaïde, Steven Van Laere, Anthony Goncalves, Patrice Viens, Daniel Birnbaum, Emilie Mamessier, Céline Callens, Davide Bedognetti

**Affiliations:** 1 Département d’Oncologie Médicale, Predictive Oncology Laboratory, Centre de Recherche en Cancérologie de Marseille (CRCM), Inserm, U1068, CNRS UMR7258, Institut Paoli-Calmettes, Aix-Marseille Université, 232, Boulevard de Sainte-Marguerite, 13009 Marseille, France; 2 Department of Medical Oncology, Institut Paoli-Calmettes, Aix-Marseille Université, Marseille, France; 3 Department of Medical Oncology, Institut Curie Saint-Cloud, Paris, France; 4 Sylvester Comprehensive Cancer Center, University of Miami, Miami, USA; 5 Department of Public Health Sciences, University of Miami, Miami, USA; 6 Center for Oncological Research (CORE), Integrated Personalized and Precision Oncology Network (IPPON), University of Antwerp, Universiteitsplein 1, Wilrijk, Belgium; 7 Tumor Biology and Immunology Laboratory, Research Branch, Sidra Medicine, Doha, Qatar

**Keywords:** Inflammatory breast cancer, Copy number alteration, Mutation, Whole-exome sequencing

## Abstract

**Background:**

Inflammatory breast cancer (IBC) is the most pro-metastatic form of BC. Better understanding of its enigmatic pathophysiology is crucial. We report here the largest whole-exome sequencing (WES) study of clinical IBC samples.

**Methods:**

We retrospectively applied WES to 54 untreated IBC primary tumor samples and matched normal DNA. The comparator samples were 102 stage-matched non-IBC samples from TCGA. We compared the somatic mutational profiles, spectra and signatures, copy number alterations (CNAs), HRD and heterogeneity scores, and frequencies of actionable genomic alterations (AGAs) between IBCs and non-IBCs. The comparisons were adjusted for the molecular subtypes.

**Results:**

The number of somatic mutations, TMB, and mutational spectra were not different between IBCs and non-IBCs, and no gene was differentially mutated or showed differential frequency of CNAs. Among the COSMIC signatures, only the age-related signature was more frequent in non-IBCs than in IBCs. We also identified in IBCs two new mutational signatures not associated with any environmental exposure, one of them having been previously related to HIF pathway activation. Overall, the HRD score was not different between both groups, but was higher in TN IBCs than TN non-IBCs. IBCs were less frequently classified as heterogeneous according to heterogeneity H-index than non-IBCs (21% *vs* 33%), and clonal mutations were more frequent and subclonal mutations less frequent in IBCs. More than 50% of patients with IBC harbored at least one high-level of evidence (LOE) AGA (OncoKB LOE 1–2, ESCAT LOE I–II), similarly to patients with non-IBC.

**Conclusions:**

We provide the largest mutational landscape of IBC. Only a few subtle differences were identified with non-IBCs. The most clinically relevant one was the higher HRD score in TN IBCs than in TN non-IBCs, whereas the most intriguing one was the smaller intratumor heterogeneity of IBCs.

**Supplementary Information:**

The online version contains supplementary material available at 10.1186/s12967-024-05198-4.

## Background

Inflammatory breast cancer (IBC) is rare (< 5% of cases), but is the most aggressive form of breast cancer due to high metastatic potential [1]. Its definition is clinical: presence of redness (occupying at least one-third of the breast) and/or oedema (“*peau d’orange*”) and/or warm breast, with or without palpable breast tumors, with rapid appearance (less than 6 months) and diagnosis of an invasive carcinoma [1]. Despite therapeutic progresses, the long-term survival remains around 50%. Better understanding of IBC pathophysiology, notably the reasons for strong aggressiveness and rapid dissemination, is crucial to develop new systemic therapies.

Its enigmatic form justified the numerous biological studies of IBC reported since several years. During the last 15 years, “omics” studies have been applied to clinical samples [2], mainly based on gene expression profiling [3]. To date, the largest series remains the one we reported within the World IBC Consortium [4, 5]. “Omics” analyses were also reported at the DNA level to define profiles of copy number alterations [6] and methylation [7], and more recently mutational profiles based on next-generation sequencing (NGS) [8–13]. The rare NGS studies of IBC tumor samples published so far are all but three based on targeted NGS (tNGS). The two largest ones, published by our groups [10, 13], reported higher tumor mutational burden (TMB) in IBCs and more frequent alteration of genes including *TP53* and genes involved in DNA repair and NOTCH pathways [10, 13]. However, no consensual molecular IBC signature emerged. Because no molecular study led to the development of specific diagnostic and/or therapeutic targets, the diagnosis of IBC remains clinical and the treatment similar to that of patients with locally advanced non-IBC.

During the last decade, whole-exome sequencing (WES) and whole-genome sequencing (WGS) of non-IBC primary tumors provided new insights into our understanding of breast cancer: identification of driver alterations, description of mutational signatures related to the mechanisms of DNA damage and DNA repair during the tumor life, and demonstration of mutational and clonal evolution with time [14–22]. Recently, such approaches were successfully applied to metastatic breast cancer samples [23–25]. To our knowledge, only two studies applied WES to clinical IBC samples [12, 26], but the series were small (respectively 22 patients with advanced HER2+ IBC and 6 patients with hormone receptor-positive (HR+) IBC) and most of patients had been pre-treated. One study applied WGS [27] to a series of 20 patients with IBC.

To fill this gap and to provide an in-depth DNA characterization of a large number of IBC, we launched WES of a multicentric retrospective series of 54 previously untreated IBC samples. Our aim was to report the mutational landscape of IBC and to search for mechanistic and/or diagnostic and/or therapeutic markers comparatively to non-IBC.

## Methods

### Patients and samples selection

In this retrospective study, all clinical samples were pre-treatment samples of primary cancers. IBC tumor samples and paired peripheral blood samples were collected from 28 patients treated at the Institut Paoli-Calmettes (Marseille, France) and 24 at the Institut Curie (Paris and Saint-Cloud, France). IBC was clinically defined as T4d according to the international consensus criteria [1]. Each patient had given written informed consent for somatic and constitutional genomic analysis. The study was approved by our institutional review boards. Extraction and quality control of tumor DNA were done as described [28]. The other selection criteria included available frozen sample, tumor cellularity (> 70%), good-quality extracted tumor DNA, available clinicopathological data and germline DNA. We also collected from The Cancer Genome Atlas (TCGA) dataset two additional IBC samples (T4d) and the non-IBC control samples (non-T4d), for which tumor and germline WES and clinicopathological data were available. Because all 54 IBC samples were from women with AJCC stage III-IV and of ductal type, and to avoid unbalance that could introduce biases in the IBC/non-IBC comparison, we selected the 102 ductal non-IBC TCGA samples from women with stage III-IV. Thus, the final series included 54 IBCs and 102 non-IBCs. The tumor molecular subtype based upon immunohistochemistry (IHC) was defined as HR+/HER2− when estrogen receptor (ER) and/or progesterone receptor (PR) expressions were positive and HER2 negative, HER2+ when HER2 was positive, and TN when the three receptors were negative. When unavailable (a few TCGA non-IBC samples), the normalized gene expression TCGA data were used to infer the receptor status as described [29].

### Whole-exome sequencing

Extraction of tumor and germline DNA from patients with IBC and quality control were done as previously described [28]. WES was performed using Illumina HiSeq 2500 sequencing systems. The 150 bp paired-end libraries were prepared using the Sureselect Human All Exome capturing kit (Agilent, Santa Clara, CA, USA) as recommended by the manufacturer. The sequence data were aligned to the human reference genome (UCSC hg19) using the Burrows-Wheeler Aligner [30]. Tumor and normal samples were sequenced at a median depth of 187× (50–463) and 63× (28–108), respectively. Bam files were deduplicated, realigned and base recalibration was applied with GATK version 3.7 [31].

Somatic single nucleotide variants (SNVs) calling were done with Mutect [32]. Somatic insertions/deletions (indels) calling were done with Strelka2 [33]. The variants, i.e., SNVs and indels, were annotated with the Annotate Variation Software (ANNOVAR, version 2013-11-12) [34]. The TCGA data were obtained from cbioportal [35]. The TMB was defined as the number of somatic coding mutations including missense, nonsense, silent, and indel divided by the panel size. Driver mutations were determined using the Cancer Genome Interpreter (CGI) [36]. COSMIC mutational signatures were computed with the python program mutation-signatures (https://github.com/mskcc/mutation-signatures). The search for other mutational signatures was done using the MutationalPatterns R-package. The regions significantly gained/amplified or lost/deleted across IBCs (q < 0.25) were identified using the GISTIC2 [37] software with the alteration threshold set at 0.2. Allelic copy number, purity and ploidy were estimated with FACETS [38]. The Homologous Recombination Deficiency (HRD) score was determined from WES data, as described [39], by considering three independent measures of genomic instability: the number of loss of heterozygosity (LOH), the number of telomeric-allelic imbalances (TAI), and the number of large-scale state transitions (LST), scored from FACETS results. The HRD score was calculated as the sum of the TAI, LST, and LOH scores, and the profile was considered HRD-high when the HRD score was ≥ 42 [40]. The Shannon’s Index (H) was used to estimate intra-tumor heterogeneity, and was assessed using the SciClone package [41]. The calculation of the Cancer Cell Fraction (CCF) for each mutation was based on the purity, allele frequency, and total copy number. Subsequently, credible intervals were computed using the bayestestR package and the Highest Density Interval (HDI) method. If the value 1 (i.e., CCF = 1, clonal) fell within the credible interval, the mutation was classified as "Clonal"; otherwise, it was categorized as "Subclonal”. Actionable genomic alterations (AGAs) were assessed following OncoKB [42] and ESCAT [43] scales.

### Statistical analysis

The continuous variables were described by median and range, and the binary variables by numbers and percentage. Correlations between tumor classes and clinicopathological or molecular variables were analyzed using the Wilcoxon test or the Fisher’s exact test when appropriate. The adjustment on molecular subtypes was done using the logit link function. Variables with p-value inferior to 0.05 were considered as significant. Analyses were done using the R-software (version 4.2.1: http://www.cran.r-project.org/).

## Results

### Patients and samples

We analyzed 54 IBC samples and 102 non-IBC TCGA samples (Additional file 1: Table S1). All cases were ductal type and from women with AJCC stage III-IV à. In the IBC group, the median patients’ age at diagnosis was 48 years (24–79) and 64% were non-menopausal; the pathological grade was 3 in 70% of cases and the molecular subtypes were HER2+ in 41% of cases, HR+/HER2− in 37%, and TN in 22%. As expected, IBCs were associated with younger patients’ age, more frequent non-menopausal status and HER2+ and TN subtypes, than non-IBCs.

### Somatic mutations

WES analysis identified 5576 somatic mutations in 4200 genes in IBCs and 6749 somatic mutations in 4839 genes in non-IBCs (Additional file 2: Table S2, Additional file 3: Table S3). The median number of somatic mutations per sample was not significantly different between both groups (62.5 in IBCs (3–942) vs. 50 in non-IBCs (4–442), p = 0.349, Wilcoxon test). The median TMB was not different between IBCs and non-IBCs (1.24 mutations/MB (0.05–21) vs. 1.32 (0.10–12) respectively, p = 0.294, Wilcoxon test; Additional file 7: Fig. S1A), even after adjustment on the molecular subtypes (p = 0.433, logit function). However, after adjustment for age differences and for molecular subtypes, the TMB was approximately 20% higher in IBC (OR = 1.18; P = 0.05). Four percent of IBC samples presented a high TMB (> 10 mutations/MB) vs. 1% of non-IBC samples (p = 0.275, Fisher’s exact test). The TMB was higher in the TNBC subtype (Additional file 7: Fig. S1B) than in the HR+/HER2− subtype in both IBCs (p = 0.115) and non-IBCs (p = 0.062). The median number of tumor neoantigens *per* sample was not significantly different between IBCs (47, range 9 to 97) and non-IBCs (34, range 3 to 574) without (p = 0.171, Wilcoxon test) and with (p = 0.963, logit function) adjustment on the molecular subtypes.

Among the somatic mutations, 96% were single nucleotide variants (SNVs) and 4% were insertions/deletions (indels) in IBCs versus 90% and 10% respectively, in non-IBCs (p = 2.21E−42, Fisher’s exact test). The difference remained significant after adjustment on the molecular subtypes (p = 8.41E−39, logit function). The percentage of non-silent mutations was lower in IBCs (74%) than in non-IBCs (78%; p = 8.99E−07, Fisher’s exact test), even after adjustment on the molecular subtypes (p = 4.28E−07, logit function). If we consider the SNVs only, the percentage of non-silent mutations was also lower in IBCs (73%) than in non-IBCs (75%; p = 4.49E−03, Fisher’s exact test), and the difference remained significant after adjustment on the molecular subtypes (p = 3.23E−03, logit function). Among the non-silent SNVs, the percentage of missenses was higher (95% *vs.* 93%) and the percentage of nonsenses was lower (5% *vs.* 7%) in IBCs than in non-IBCs (p = 2.74E−04, Fisher’s exact without adjustment for the molecular subtypes and p = 4.13E−04, logit function with adjustment).

**Fig. 1:**
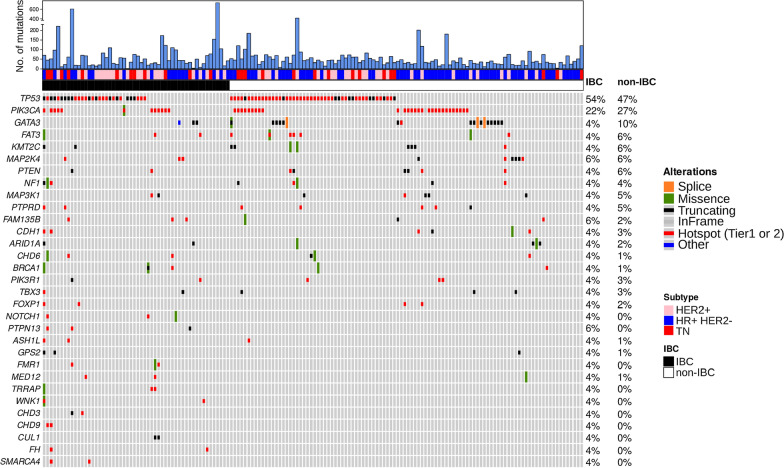
Distribution of alterations of the top 31 genes mutated in IBCs. Oncoprint of the top 31 genes mutated in at least two IBC samples. *Top*: Number of smatic mutations in each sample. IHC-based molecular subtypes and IBC/non-IBC groups are color-coded as indicated in the legend. *Bottom*: somatic gene mutations color-coded according to the legend. The genes are ordered from top to bottom by decreasing percentage of altered IBCs right panel). The percentages of mutation in IBCs and non-IBCs are shown to the right of the Oncoprint

A total of 195 out of 5576 mutations (3.5%) were defined as driver mutations (TIER1-TIER2) by Cancer Genome Interpreter (CGI) in IBCs *versus* 357 (5%) in non-IBCs (Wilcoxon test: p = 4.60E-02 without adjustment for the molecular subtypes, but p = 0.829 after adjustment). They concerned 117 genes in IBCs (Additional file 2: Table S2), including classical driver genes of breast cancer, such as *TP53*, *PIK3CA*, *MAP2K4*, *GATA3*, or *KMT2C*. The 31 genes mutated in at least 2 IBCs are shown in Fig. 1. Forty-eight percent of them are included in the 93-gene list of driver genes defined by Nik-Zainal et al. in non-IBCs [20]. The most commonly mutated gene was *TP53* (54% of samples), followed by *PIK3CA* (22%). All 117 genes displayed similar mutation frequency between IBCs and non-IBCs in our series (p > 0.05; Fisher’s exact test).

### Mutational spectra and processes of somatic SNVs

**Fig. 2:**
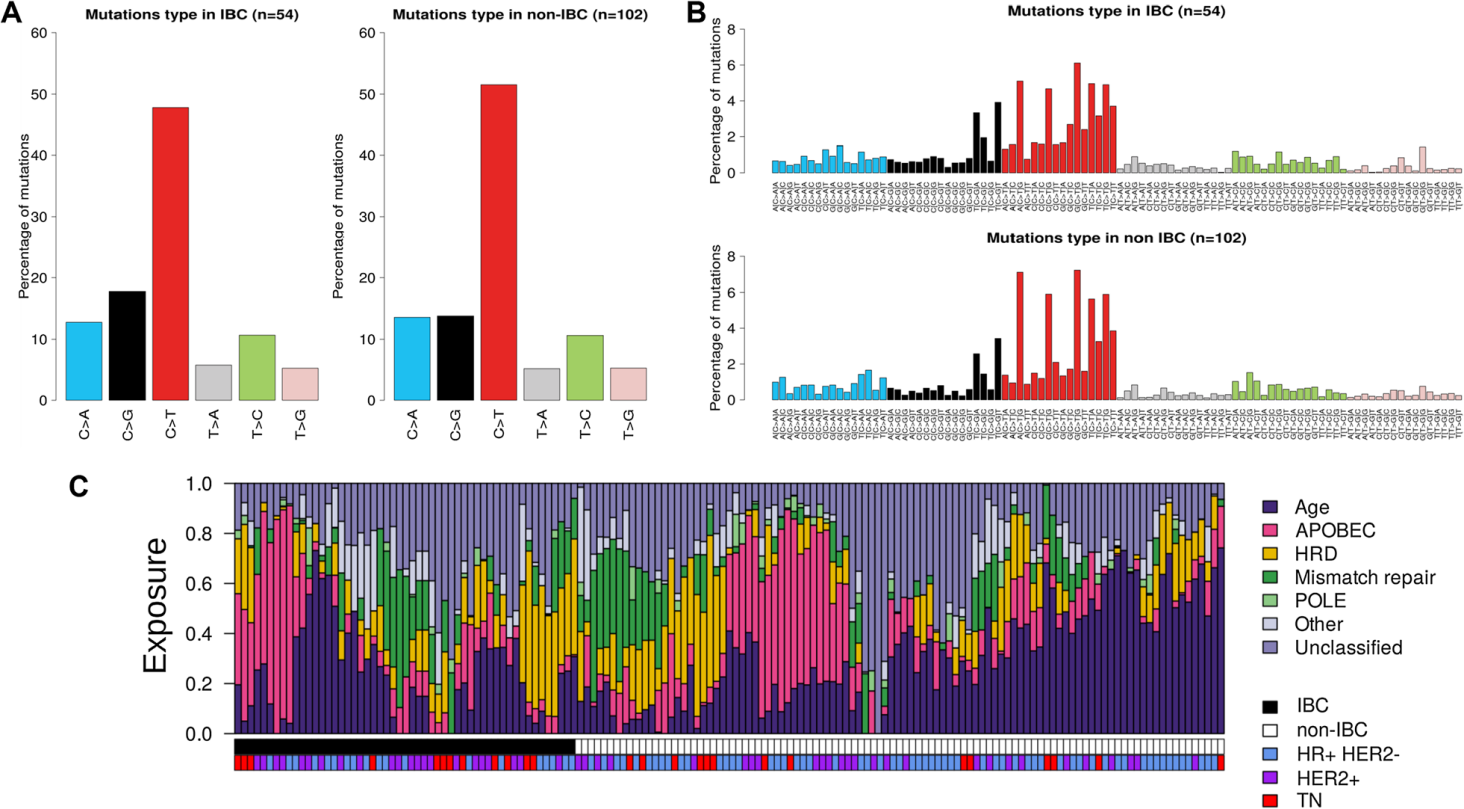
Mutational processes of somatic SNVs in IBCs. **A** Proportions of base substitutions with respect to single-nucleotide-mutation contexts in IBCs and non-IBCs. **B** Similar to **A** but with respect to tri-nucleotide mutation contexts. **C** Proportions of the most represented COSMIC mutational signatures in the whole population age-related: signature 1; homologous recombination deficiency HRD: signature 3; APOBEC activation: signatures 2 and 13; mismatch repair: signatures 6, 20 and 26; POLE: signature 10). The signatures, IHC-based molecular subtypes and IBC/non-IBC groups are color-coded according to the legend

The proportions of base substitutions across SNVs are shown in Fig. 2A, B. In IBCs, the most frequent base change was C > T (average of 48% of substitutions) with respect to single-nucleotide-mutation contexts (Fig. 2A), as observed in non-IBCs (average of 52%). The mutational spectra were similar between IBCs and non-IBCs, except an enrichment in C > G in IBCs (average of 18% vs 14%, p = 2.6E−02, Wilcoxon test; and p = 0.070 after adjustment for the molecular subtypes). The same analysis regarding the tri-nucleotide mutation contexts (Fig. 2B) showed that the most frequent base change in IBCs was G[C > T]G, as observed in non-IBCs; the comparison between IBCs and non-IBCs revealed an enrichment in 11 substitutions and tri-nucleotide contexts in IBCs, notably the G[C > G]G (p = 1.01E−03, Wilcoxon test) and the G[C > T]C (p = 1.42E−02, Wilcoxon test), but none remained significant after FDR correction.

We then assessed the distribution of the 30 COSMIC mutational signatures. In both IBCs and non-IBCs, the most represented signatures were, as expected, the signatures 1 (age-related), then 2 and 13 (APOBEC activation), then 3 (homologous recombination deficiency), and then 6, 20 and 26 (mismatch repair) (Fig. 2C). In IBC, positive correlations existed between the abundances of signature 2 (Additional file 8: Fig. S2A) and of signature 13 (Additional file 8: Fig. S2B) and higher TMB (p = 4.0E−04 and p = 5.6E−03 respectively, Wilcoxon test), and between the abundances of signature 3 (Additional file 8: Fig. S2C) and higher HRD score (p = 1.2E−03, Wilcoxon test), The comparison with non-IBCs identified only one signature differentially represented between both groups: the signature 1 was less frequent in IBCs than in non-IBCs (p = 1.69E−02, Wilcoxon test), even after adjustment on molecular subtypes (p = 3.91E−02, logit function; Fig. 2C).

Given the enrichment in C > G transversions in IBC, we set out to identify possible new mutational signatures. Using non-negative matrix factorization (NMF), six signatures were extracted from our data. Four of these were associated with mutational processes associated with APOBEC activity (N = 2), mismatch repair (N = 1), and homologous recombination deficiency (N = 1). The two remaining signatures (i.e. SBSA and SBSB) have an unknown etiology and were not recovered from the non-IBC mutational profiles. SBSA is characterized by a rather flat profile, whereas SBSB shows dominance of C > T transitions (Additional file 8: Fig. S2D).

### Copy number alterations

**Fig. 3:**
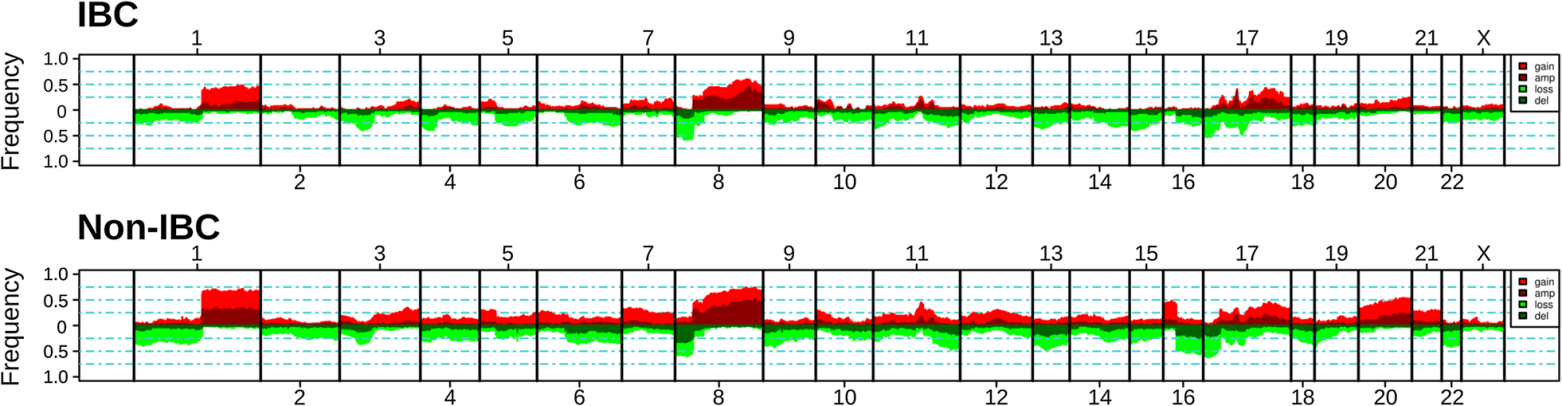
Frequency plots of CNAs in IBCs. Frequencies vertical axis, from 0 to 100%) are plotted as a function of chromosome location for IBCs *top*) and non-IBCs *middle*). Vertical lines indicate chromosome boundaries. The CNAs are color-coded as indicated in the legend: gains red), amplifications dark red), losses green), and deletions dark green)

Figure 3 shows the frequency plots of low- or high-level CNAs. In non-IBCs, the most frequently gained regions were on 1q, 8q, 11q, 17q and 20q chromosomal arms, whereas the regions frequently lost were on 8p, 11q and 16q. Globally, visual inspection did not reveal obvious differences between IBCs and non-IBCs in terms of altered regions and of frequencies of alterations. GISTIC analysis of IBC samples identified 28 chromosomal cytobands significantly (q < 0.25) gained/amplified (total length of 92 Mb) and 18 chromosomal cytobands significantly (q < 0.25) lost/deleted (total length of 854 Mb). The gained/amplified cytobands comprised 725 genes including 8 defined as driver alterations by CGI: *HER2*, *CCND1*, *MYC*, *EGFR*, *PIK3CA*, *FGFR2*, *MDM4*, and *AKT3*, as well as *FGFR1, ZNF703.* The two most significant gained/amplified cytobands (17q12 and 11q13.3) were regions classically amplified in breast cancer. As expected, all HER2-negative IBC tumors (by IHC/FISH) had no *HER2* gain/amplification, whereas 19 out of 22 HER2-positive IBC tumors had *HER2* gain/amplification. The three discordant tumors have no *HER2* mutation and likely represent false negatives probably due to sampling bias such as normal tissue contamination or tumor heterogeneity. The lost/deleted cytobands comprised 6,427 genes, including 25 identified as driver genes by CGI, such as *NF1*, *TP53*, *CDKN1A*, *ATM, STK11, BAP1, ARID1A* (Additional file 4: Table S4). We compared the alteration frequencies between IBCs and non-IBCs of genes included in the GISTIC regions gained/amplified in IBCs (Additional file 5: Table S5) and of genes included in the GISTIC regions lost/deleted in IBCs (Additional file 6: Table S6). No gene was more frequently gained/amplified in IBCs than in non-IBCs. Thirty-seven genes (located on 8q21 and including *IL7* and *HEY1*) were more frequently lost/deleted in IBCs than in non-IBCs (p < 0.05; Fisher’s exact test), but none of them remained significant after FDR correction (p > 0.5).

### Genomic complexity

**Fig. 4:**
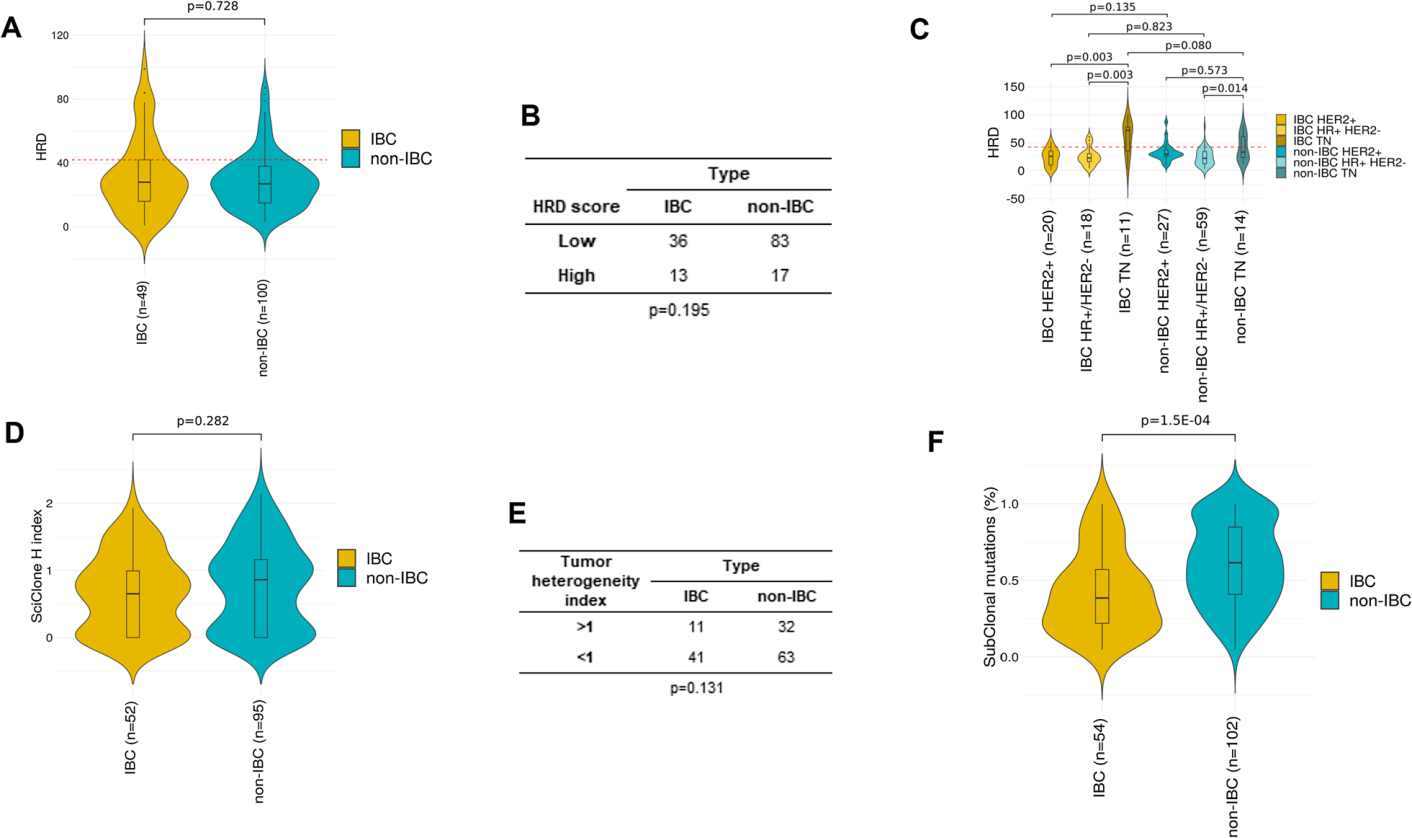
HRD score, heterogeneity index and mutational clonality in IBCs. **A** Box-plot of HRD score in non-IBC and IBC samples. **B** Contingency table between HRD score and IBC/non-IBC groups. **C** Similar to A/, but per molecular subtype. **D** Box-plot of Heterogeneity H) index in non-IBC and IBC samples. **E** Contingency table between the tumor heterogeneity status and IBC/non-IBC groups. **F** Box-plot of the percentages of clonal and subclonal mutations in non-IBC and IBC samples

The HRD score was not different between IBCs (median = 28, range 1–99) and non-IBCs (median = 27, range 3–87; p = 0.728, Wilcoxon test; Fig. 4A). By using the classical positivity cut-off (score = 42), 27% of IBC samples were defined as “BRCAness” *versus* 17% of non-IBC samples, but the difference was not significant (p = 0.195, Fisher’s exact test; Fig. 4B). As expected, this HRD score was higher in the TN subtype than in the HR+/HER2− subtype in both IBCs (p = 0.014) and non-IBCs (p = 3.0E−03, Wilcoxon test; Fig. 4C). Interestingly, it also tended to be higher in TN IBCs than in TN non-IBCs (p = 0.08, Wilcoxon test).

We also measured the intratumor heterogeneity of tumor samples using SciClone. Heterogeneity index (H-index) was slightly lower in IBCs (median = 0.65, range 0–1.92) than in non-IBCs (median = 0.86, range 0–2.13) samples (p = 0.282, Wilcoxon test; Fig. 4D). Twenty-one percent of IBCs (11/52) *vs* 33% (32/95) of non-IBCs displayed an H-index superior to 1 (OR = 1.88; p = 0.131, Fisher’s exact test), corresponding to more heterogeneous tumors than the ones with an H-index inferior to 1. Finally, we assessed the percentage of clonal or subclonal non-synonymous mutations in all samples (Fig. 4F). The proportions of clonal mutations were higher in IBCs than in non-IBCs (p = 1.4E−04, Wilcoxon test; p = 3.0E−04 after adjustment upon the molecular subtypes), and consequently subclonal mutations were more frequent in non-IBCs. In IBCs, a positive correlation existed between the abundance of APOBEC signature 13 (Additional file 8: Fig. S2E) and that of subclonal mutations (p = 0.217, Wilcoxon test). When assessing the number of tumor neoantigens in function of the clonality of non-synonymous mutations, we did not observe significant differences in terms of clonal neoantigens (p = 0.980) but the number of subclonal neoantigens tended to be lower in IBC (p = 0.098). In line with this, tumor neoantigens are significantly more often subclonal in non-IBC (p = 0.016), but not in IBC (p = 0.455).

### Actionable genetic alterations

**Fig. 5:**
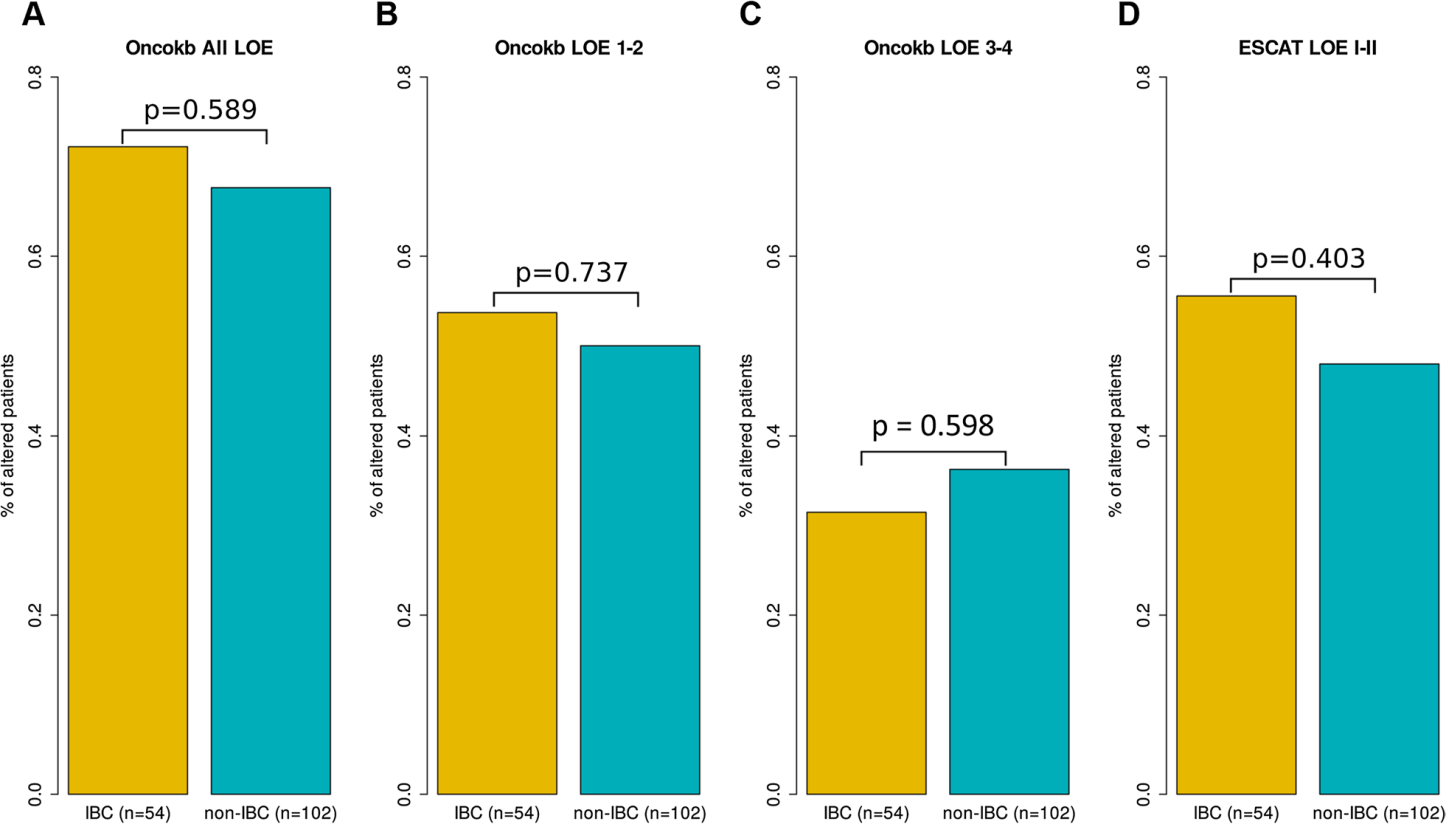
Percentages of patients with AGAs in IBCs. **A** Bar-plots of the percentages of patients with IBC and non-IBC displaying at least one OncoKB AGA. The p-value is for the Fisher’s exact test. **B** Similar to **A**, but for OncoKB LOE 1–2 AGAs. **C** Similar to **A**, but for OncoKB LOE 3–4 AGAs. **D** Similar to **A**, but for ESCAT LOE I–II AGAs

Using the OncoKB database of actionable genetic alterations (AGAs) [42], two levels of clinical evidence (LOE) were distinguished: LOE 1–2 corresponding to standard care therapies, and LOE 3–4 corresponding to investigational therapies. Overall, 72% of IBC samples (39/54) had at least one AGA, *versus* 68% of non-IBC samples (69/102) when considering all LOE pooled (p = 0.589, Fisher’s exact test; Fig. 5A). Regarding the LOE 1–2 AGAs, 54% of IBCs (29/54) displayed at least one alteration *versus* 50% of non-IBCs (51/102; p = 0.737, Fisher’s exact test; Fig. 5B). In IBCs, the LOE 1–2 alterations included *HER2* amplifications (35%; 19 patients), *PIK3CA* mutations (22%; 12 patients), and *BRCA1* mutation or deletion (6%; 3 patients). For LOE 3–4 AGAs, these figures were 31% in IBCs (17/54) and 36% in non-IBCs (37/102; p = 0.598, Fisher’s exact test; Fig. 5C). Of note, 12/54 IBC samples (22%) had two or more AGAs simultaneously, suggesting potential interest of drug combinations, including two patients with double level 1 *PIK3CA* mutation. The same was observed in non-IBCs, with 27/102 samples (26%) having two or more AGAs, including 2 patients with double level 1 *PIK3CA* mutation. The same analysis was done using the ESCAT LOE I–II AGAs. Fifty-five percent of IBCs (30/54) displayed at least one alteration *versus* 48% of non-IBCs (49/102; p = 0.403, Fisher’s exact test; Fig. 5D). In IBCs, the identified ESCAT LOE I AGAs included *HER2* amplification (35%; 19 patients), and *PIK3CA* mutations (19%; 10 patients). No germline *BRCA1/2* mutation, nor MSI status, nor *NTRK* fusion were identified. The ESCAT LOE II AGAs included *PTEN* deletion (6%; 3 patients) and *AKT1* mutation (2%; 1 patient). No other ESCAT II alteration (*ESR1* mutation, *HER2* mutation) was identified. In both IBC and non-IBC, profiles of AGAs in *PIK3CA* and *ERBB2* AGAs appeared to be mutually exclusive, although our sample size is too limited to obtain statistical significance.

## Discussion

We compared the WES profiles of untreated primary tumors of 54 IBCs and 102 non-IBCs. To our knowledge, this is by far the largest series of IBCs profiled with WES.

To avoid DNA biases induced by previous DNA-damaging treatments or resistance mechanisms [23, 44], we analyzed untreated primary tumors only. Because of unbalance between IBCs and non-IBCs regarding AJCC stages and molecular subtypes, we selected stage III-IV non-IBC controls and adjusted the comparisons upon the molecular subtypes. For each patient, normal DNA was sequenced. In addition to the higher number of IBC samples, these points distinguish our study from the previous WES/WGS studies. In the first WES study, the authors profiled 22 HER2+ IBC biopsies collected in the metastatic setting, and after chemotherapy in 69% of cases and trastuzumab in 32% [12], 22 paired germline DNA, and used 131 TCGA HER2+ non-IBCs as comparators. In the second WES study, Luo et al*.* profiled 6 HR+ IBC samples collected at time of surgery, after NACT, but did not sequence the germline DNA, nor compared with non-IBCs [26]. In the WGS study [27], 20 IBC biopsies, likely pre-chemotherapy, were profiled, with corresponding germline DNA, and 23 molecular subtype- and TCGA stage-matched non-IBCs served as controls. Of note, the small number and the type of IBC samples profiled through these studies clearly point the difficulty of profiling a pre-treatment tumor sample in such a rare cancer.

The number of somatic mutations and TMB were not different between IBCs and non-IBCs. This is consistent with the results of the WGS study [27], but in contrast with previous tNGS-based studies and the Goh et al. WES study that reported higher TMB in IBCs. However, in these studies, the sequencing depth of non-IBC controls (average depth close to ~ 100 × with TCGA) was inferior to the sequencing depth of IBCs, possibly explaining the lower TMB in non-IBCs. Another explanation for the absence of TMB differences in the current series relates to age differences between IBC and non-IBC cases. As cancer genomes of older patients are characterized by higher frequencies of mutations, age differences between two patient cohorts may obscure biologically relevant differences. Indeed, age-adjusted TMB was approximately 20% higher in IBC as compared to non-IBC. As reported in the WGS study [27], we found no gene significantly differentially mutated in its coding sequences between both groups. Classical driver genes were mutated in IBCs, the most frequent being *TP53* as tumor suppressor gene and *PIK3CA* as oncogene. The only and very subtle significant differences were a higher percentage of SNVs (*vs* indels), a smaller percentage of non-silent mutations (*vs* silent), and a higher percentage of missenses (*vs* nonsenses) in IBCs than in non-IBCs.

In term of mutational spectra, the most frequent base change in IBC was C > T (48% of substitutions), in agreement with previous reports in non-IBCs [20, 45] and IBCs [26]. Mutational spectra were similar between IBCs and non-IBCs, except an enrichment in C > G in IBCs and in several substitutions in several trinucleotide contexts, suggesting a distinct and not previously identified mutational signature in IBC. To further explore the mutational processes shaping the IBC landscape, we assessed the proportions of COSMIC mutational signatures [45]. In IBCs, like in non-IBCs, the most represented ones were the age-related signature (S1), followed by APOBEC activation signatures (S2, S13), then HRD signature (S3). As expected, the abundances of APOBEC signatures positively correlated with TMB and abundances of subclonal mutations [46], whereas the abundance of signature 3 positively correlated with HRD score. The IBC/non-IBC comparison identified only one signature differentially represented: the age-related signature (S1) was more frequent in non-IBCs than in IBCs even after adjustment on molecular subtypes. Such enrichment, which agrees with the older age of patients with non-IBC when compared with IBC, validates our WES data but does not bring any new information. Therefore, we explored to possible existence of new mutational signatures in IBC, and identified six signatures of which two were unique to IBC. One of them, SBSB, is characterized by C > T transitions in a GpCpN or TpCpN context, previously related to HIF pathway activation [47]. The second one, SBSA, has a rather flat pattern with no obvious discernable feature. None of these signatures can be associated with any of the signatures of mutational processes (COSMIC) or environmental exposure [48]. As our data set is small, further research into these mutational signatures is needed before definitive conclusions can be drawn.

GISTIC analysis of IBCs identified 28 recurrently gained/amplified regions and 18 recurrently lost/deleted regions, including regions classically altered in BC, such as 17q12 or 8p11.23 (gained) and 6q26 or 8p21.3 (lost). The most frequently gained/amplified regions in IBCs (8q24 and 17q12) were the same as those identified previously by our team [6]. The most frequently most/deleted regions in IBCs (> 50% of cases) were located on 8p chromosome arm, including genes encoding for IL7 (i.e. cytokine involved in T- and B-cell maturation) and HEY1 (i.e. transcription factor involved in the Notch signaling pathway). None of the 6,427 genes located in GISTIC regions was differentially altered between IBCs and non-IBCs in our series after FDR correction.

The HRD score was not different between IBC and non-IBC samples. This does not confirm our previous study [13] in which the HRD score, measured on array-CGH, was higher in IBCs. This difference may rely on disease heterogeneity, but also on the use of different HRD scores: the score used here, based on WES data, includes three components (LOH, TAI, LST), whereas the score measured from array-CGH was only based on LOH associated with DNA loss. However, the present HRD score tended to be higher in TN IBCs than in TN non-IBCs (p = 0.08), the difference being not significant likely because of the small number of samples (12 IBCs, 15 non-IBCs). This requires validation in larger series, and suggests that TN IBCs might be more sensitive to PARP inhibitors than TN non-IBCs.

Intratumor heterogeneity is a prevalent feature in many cancer types and represents a considerable challenge to optimizing prognosis and treatment. Heterogeneity H-index was slightly lower in IBCs than in non-IBCs, and IBCs were less frequently classified as heterogeneous than non-IBCs (21% vs 33%). This difference only tended towards significance, likely because of the relatively small series size. It is consistent with the WGS study that reported more clonal tumors among IBCs than non-IBCs [27]. Accordingly, we found more clonal mutations and less subclonal mutations in IBCs than in non-IBCs. This suggests that IBCs might be more homogeneous than non-IBCs, an observation rather counter-intuitive given the higher aggressiveness of IBCs. A possible explanation might be the more rapid proliferation rate and clinical evolution of IBCs as compared to stage III-IV non-IBCs that evolve more slowly during several years. Interestingly, although the number of tumor neoantigens per sample is not different between IBC and non-IBC, we observed a lower number of subclonal neoantigens per sample in IBC. Based on these observations, we hypothesize that tumor neoantigen bearing subclones in IBC may be efficiently eradicated by a potent inflammatory response, hence also explaining the typical symptoms of the disease. At present, this statement is speculative but it deserves further investigation.

To define AGAs, we used the OncoKB and ESCAT classification systems as recommended by ESMO Precision Medicine Working Group [49]. Both systems identified a similar percentage of patients with AGA in IBC and non-IBC groups. Regarding the clinically relevant AGAs, more than 50% of patients with IBC harbored at least one alteration (OncoKB LOE 1–2: 54%, ESCAT LOE I–II: 55%). This percentage is similar to the 54% rate we previously reported in another clinical series using tNGS, array-CGH and another AGA classification system [13]. Inclusion of OncoKB LOE 3–4 alterations further increased this percentage to 72% in patients with IBC vs 68% in patients with non-IBC. Such an elevated percentage, which would further be increased by incorporating complex genomic scores (e.g. HRD, TMB, MSI status), suggests that genomics-based precision medicine deserves evaluation in IBC.

We acknowledge a few limitations to our study: retrospective nature and associated biases, and no analysis of structural variations, nor gene fusions. However, it displays several strengths: largest WES study of IBCs, consensual definition for IBCs, previously untreated primary tumors, profiling of matched normal samples, comparison with AJCC stage III-IV non-IBC samples, similar sequencing platforms and depth for both groups, adjustment upon the molecular subtypes, statistical correction (FDR with q-values) for multiple tests in the comparative analyses; and consensual AGAs definitions.

## Conclusions

We present the largest WES landscape of untreated IBC clinical tumor samples. We could not identify any significant genomic alteration different between IBCs and non-IBCs, notably regarding the mutational and CNA profiles, TMB and HRD scores, and presence of AGAs. A few subtle differences were identified: higher HRD score in TN IBCs versus non-IBCs, smaller intratumor heterogeneity in IBCs than in non-IBCs with more clonal but less subclonal mutations. These relatively subtle mutational differences at the tumor bulk level require validation in independent and larger series, and call for additional analysis levels both in term of clinical samples (tumor emboli, tumor microenvironment, blood,…) and technologies (spatial transcriptomics, single-cell profiling, epigenomics, metabolomics,…).

### Supplementary Information

**Additional file 1: Table S1.** Clinicopathological characteristics of patients and samples.

**Additional file 2: Table S2.** List of somatic mutations in IBCs.

**Additional file 3: Table S3.** List of somatic mutations in non-IBCs.

**Additional file 4: Table S4.** List of gained/amplified and lost/deleted regions according to the GISTIC analysis of CNAs in IBC.

**Additional file 5: Table S5.** Comparison of alteration frequencies between IBC and non-IBC of genes included in the GISTIC regions gained/amplified in IBCs.

**Additional file 6: Table S6.** Comparison of alteration frequencies between IBC and non-IBC of genes included in the GISTIC regions lost/deleted in IBCs.

**Additional file 7: Figure S1.** Tumor mutational burden TMB) in IBCs and non-IBCs. A/ Violin plots showing the distribution of the TMB in IBCs and non-IBCs. The p-value is for the Wilcoxon test. B/ Similar to A/ but *per* molecular subtype.

**Additional file 8: Figure S2.** Correlations between APOBEC and HRD signatures and TMB and HRD score in IBCs. A/ Box plots showing the distribution of the TMB according to the abundance of APOBEC signature 2 Neg: patients with signature < 10%; Pos: patients with signature > 10%). The p-value is for the Wilcoxon test. B/ Similar to A/, but according to the abundance of APOBEC signature 13. C/ Box plots showing the distribution of the HRD score according to the abundance of HRD signature 3 Neg: patients with signature < 10%; Pos patients with signature > 10%). The p-value is for the Wilcoxon test. D/ Two mutational signatures of unknown etiology SBSA and SBSB) identified in IBC samples. SBS-A is characterized by a rather flat profile, whereas SBS-B shows dominance of C > T transitions in a GpCpN or TpCpN context. E/ Box plots showing the distribution of the percentage of subclonal mutations according to the abundance of APOBEC signature 13 Neg: patients with signature < 10%; Pos: patients with signature > 10%). The p-value is for the Wilcoxon test.

## Data Availability

All clinicopathological data and genomic data analyzed in the present study will be available after paper acceptance upon reasonable request.

## Abbreviations

aCGH: Array-comparative genomic hybridization
AGA: Actionable genetic alteration
AJCC: American Joint Committee on Cancer
CNA: Copy number alteration
HR+: Hormone receptor-positive
HRD: Homologous recombination deficiency
IBC: Inflammatory breast cancer
IHC: Immunohistochemistry
Mb: Megabase
NGS: Next-generation sequencing
non-IBC: Non-inflammatory breast cancer
SNV: Single nucleotide variant
TCGA: The Cancer Genome Atlas
TMB: Tumor mutational burden
TN: Triple-negative
tNGS: Targeted next-generation sequencing
WES: Whole-exome sequencing
WGS: Whole-genome sequencing
