# Supplementary figures and images for: Mutational landscape of inflammatory breast cancer
Source: J Transl Med. 2024 Apr 18;22:374. doi: 10.1186/s12967-024-05198-4 (PMC11025259; doi:10.1186/s12967-024-05198-4)

## Slide 1
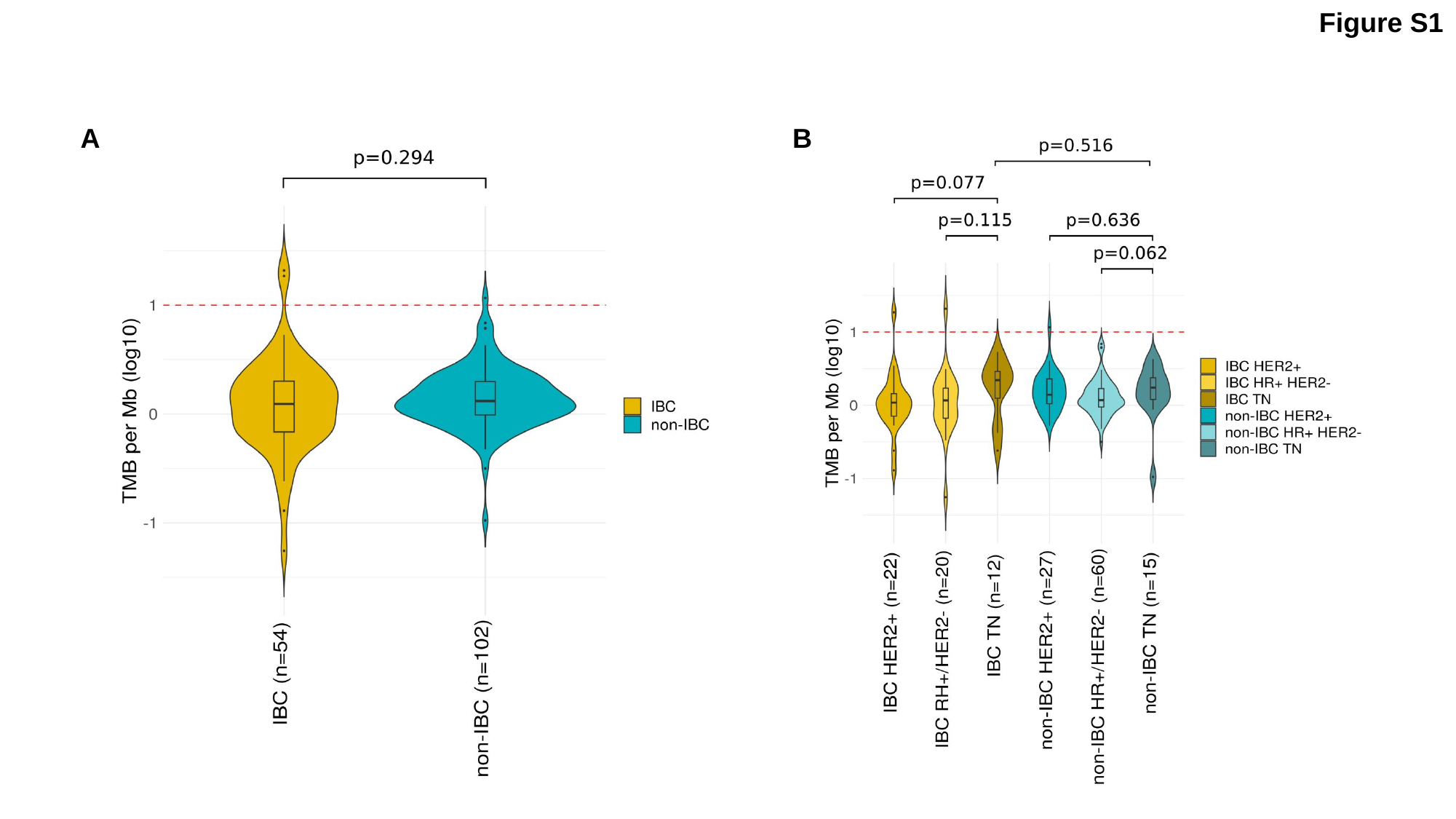

Figure S1
A
B

Supplement: Supplementary file 7 — Additional file 7: Figure S1. Tumor mutational burden TMB) in IBCs and non-IBCs. A/ Violin plots showing the distribution of the TMB in IBCs and non-IBCs. The p-value is for the Wilcoxon test. B/ Similar to A/ but per molecular subtype. [file 12967_2024_5198_MOESM7_ESM.pptx]

## Slide 1
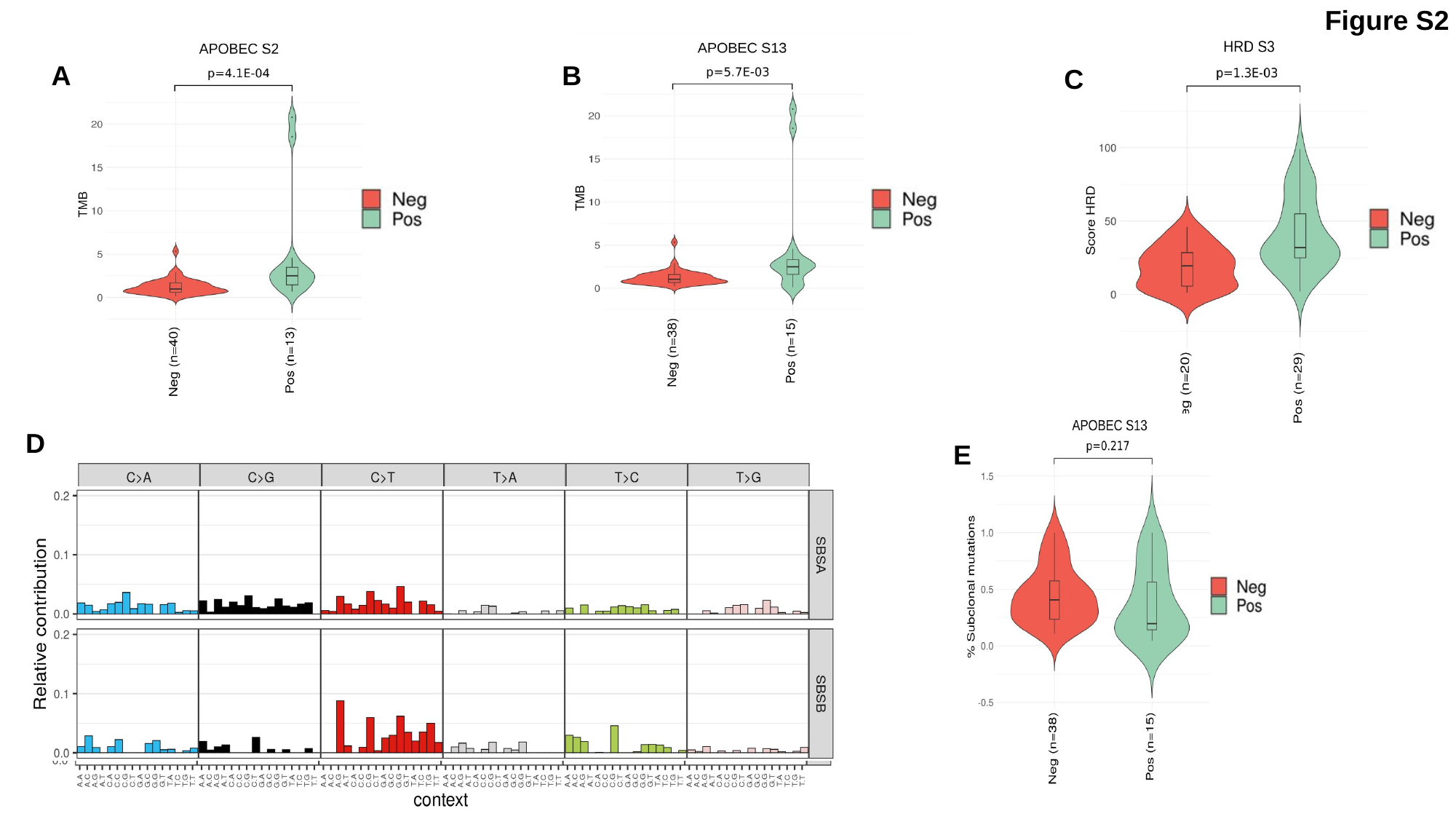

Figure S2
B
C
A
E
D

Supplement: Supplementary file 8 — Additional file 8: Figure S2. Correlations between APOBEC and HRD signatures and TMB and HRD score in IBCs. A/ Box plots showing the distribution of the TMB according to the abundance of APOBEC signature 2 Neg: patients with signature < 10%; Pos: patients with signature > 10%). The p-value is for the Wilcoxon test. B/ Similar to A/, but according to the abundance of APOBEC signature 13. C/ Box plots showing the distribution of the HRD score according to the abundance of HRD signature 3 Neg: patients with signature < 10%; Pos patients with signature > 10%). The p-value is for the Wilcoxon test. D/ Two mutational signatures of unknown etiology SBSA and SBSB) identified in IBC samples. SBS-A is characterized by a rather flat profile, whereas SBS-B shows dominance of C > T transitions in a GpCpN or TpCpN context. E/ Box plots showing the distribution of the percentage of subclonal mutations according to the abundance of APOBEC signature 13 Neg: patients with signature < 10%; Pos: patients with signature > 10%). The p-value is for the Wilcoxon test. [file 12967_2024_5198_MOESM8_ESM.pptx]
